# Protective effects of vitamin E and *Cornus mas* fruit extract on methotrexate-induced cytotoxicity in sperms of adult mice

**Published:** 2014

**Authors:** Leila Zarei, Rajabali Sadrkhanlou, Rasoul Shahrooz, Hassan Malekinejad, Behroz Eilkhanizadeh, Abbas Ahmadi

**Affiliations:** 1 *Department of Basic Sciences, Faculty of Veterinary Medicine, Urmia University, Urmia, Iran; *; 2 *Department of Pathology, Faculty of Medicine, University of Medical Sciences, Urmia, Iran.*

**Keywords:** *Cornus mas*, Methotrexate, Mouse, Sperm quality, Vitamin E

## Abstract

This study was aimed to assess the protective effects of *Cornus mas* fruit extract (CMFE) and vitamin E (Vit E) on sperm quality parameters in the methotrexate (MTX)-treated mice. Forty-eight young adult male mice (8-12 weeks) were randomly divided into six groups including control and test groups. The control group received normal saline orally , and the test groups were treated MTX (20 mg kg^-1^, ip, once weekly), MTX + CMFE (250 mg kg^-1^), MTX + CMFE (500 mg kg^-1^), MTX + CMFE (1000 mg kg^-1^), and MTX + Vit E (100 IU kg^-1^, po) for 35 consecutive days. On day 35, after euthanasia the epididymal sperms were isolated. Then the total mean sperm count, sperm viability and motility were determined. The total antioxidant capacity (TAOC) of all experimental groups were also evaluated. The MTX-treated animals showed a significant changes in all parameters of sperm quality assessment compared to the control group. Both Vit E and CMFE were able to protect from MTX-induced effects on sperm maturity and DNA damage. Co-administration of MTX and CMFE and/or Vit E resulted in protection from MTX-reduced TAOC. In conclusion, these data suggested that MTX administration could adversely affect the sperm quality. Moreover, the protective effect of Vit E and CMFE on MTX-induced sperm toxicity was also documented.

## Introduction

Chemotherapy includes use of the chemicals to prevent growth of and removing cancer cells even in the sites far away from the initial tumor origin. However, this therapeutic process cannot differentiate between cancer cells and healthy ones and along with elimination of cancer cells it targets the healthy and other rapid growing cells including blood cells, hair, and epithelial cells of digestive system.^   1 ^ Methotrexate, a folic acid antagonist, was introduced in 1950 as an effective anticancer compound in chemotherapy.^   2 ^ It is widely used in treating neoplastic disorders, leukemia, breast cancer, and testis tumors.^3^^,^^4^ This compound is also used for treating psoriasis, arthritis rheumatoid, and autoimmune disease.^5^^,^^6^ Methotrexate competitively and irreversibly prevents activity of dihydrofolate reductase (DHFR), an enzyme that participates in synthesis of tetrahydrofolate.^7^ This enzyme catalyzes transformation of dihydrofolate into active tetrahydrofolate. Affinity of MTX to DHFR is 1000 times more than folate.^8^ Since folic acid is necessary for anabolism of nucleotides, MTX prevents the synthesis of nucleotides through reaction with DHFR and subsequently prevents synthesis of DNA and RNA.^9^^,^^10^ Most chemotherapy agents act through reaction with DNA or its precursors preventing synthesis of new genetic materials. Damage to the genetic materials causes disorder in functions of somatic and reproductive cells.^11^^-^^13^ Toxic effects on reproductive cells may result in fetal imparities, changes in endocrine function, reproductive problems, and premature abortion.^14^^,^^15^ Therefore, along with MTX's therapeutic capacity, its toxic side effects appear in organs including gastrointestinal system, angiogenic system, and nervous system.^16^^-^^18^ Although mechanisms of tissue damage of testis due to this agent are not precisely known, several studies have shown that methotrexate causes biochemical stresses via disruption of oxidation-reduction equilibrium which can result in extensive physiological disorders. It was reported that Vitamin E (Vit E) as a well-known antioxidant agent exerts its protective effect in removing oxidative stress in the testis of the mice.^19^ *Cornus mas* fruit extract is a member of *Cornacea* species. In traditional medicine *Cornus mas* has been used as an herbal medicine for treating inflammation.^20^ Chemical analysis of CMFE has shown that it is a rich source of antioxidant and phenolic compounds. In addition, it contains some vitamins including vitamin C, B_1_, B_2_, E, and anthocyanins, flavonoids, and very high levels of oxalic acid.^21^^,^^22   ^ The current study was designed to assess the antioxidant effect of CMFE and Vit E in preventing toxicity of MTX influencing sperm quality parameters. 

## Materials and Methods

**Chemicals.** Vitamin E from Behsa Pharmaceutical Co. (Tehran, Iran) and methotrexate sodium manufactured by Kocak Farma Ltd. (Istanbul, Turkey) were used as the test substances.

**Preparation of alcoholic extract of** ***Cornus mas*** **fruit. **After removing of *cornus mas* stone the fruit was dried in open air, protected from direct sunlight, and then powdered. The powder was kept in a closed box at 8 ˚C. Five hundred grams of the powder was extracted using mixture of ethanol:water (7:3) at 25 ± 2 ˚C. The solvent was completely removed by rotary vacuum evaporator at 50 ˚C. The extract was freeze-dried and stored in a vacuum desicator until use.^23^

**Animals and treatment groups.** Forty-eight young (8-12 weeks) male NMRI mice were used in this study. In the beginning, the animals were kept for one week in standard conditions including temperature 22 ± 2 ˚C, 30 to 60% of humidity and the illumination cycles of 14 hr light and 10 hr darkness. Food and water were freely accessible to them. In control group (1), only normal saline was injected without chemotherapy. Mice in group 2 were injected MTX 20 mg kg^-1^ per week for 5 weeks, intraperitoneally,^   24 ^ in groups 3, 4 and 5 along with MTX (20 mg kg^-1^) received oral CMFE with daily dose of 1000, 500 and 250 mg kg^-1^.^21^^,^^23^ In group 6, animals received oral Vit E 100 UI kg^-1^ daily along with MTX (20 mg kg^-1^).^   25 ^ After 35 days, the mice were anesthetized with 25 mg kg^-1 ^ketamine (Alfasan, Utrecht, The Netherlands) for blood sampling and they were euthanized with ketamine over dose (100 mg kg^-1^). Then, both epididymis (cauda and vas) of each mouse were transferred to a 60 mm Petri dish containing 1 mL Human Tubal Fluid (HTF; Sigma, St. Louis, USA) culture and 4 mg mL^-1^ bovine serum albumin (BSA; Sigma, St. Louis, USA) medium pre-warmed to 37 ˚C. The caudate was minced making 5 to 7 slashes with a 30-gauge needle of an insulin syringe. After 30 min incubation at 37 ˚C in 5% CO_2_, spermatozoa released from epididymis.^   26 ^

**Total antioxidant capacity measurement. **The TAOC of serum from all groups was measured. This test was based on the assessment of ferric reduction antioxidant power (FRAP).^   27 ^ Briefly, at low pH, which was achieved by addition of acetate buffer (300 mM, pH 3.6), reduction of ferric tripyridyltriazine complex to the ferrous form produces an intensive blue color that could be measured at 593 nm. Aqueous solution of ferrous sulfate and appropriate concentrations of freshly prepared ascorbic acid were used as blank and standard solutions, respectively. The protein content of the samples was measured according to the Lowry method.^   28 ^

**Assessment** **of** **sperm** **count. **In order to count sperms, a 1:20 dilution was prepared. In a 1 mL microtube 190 µL of distilled water was poured and 10 µL of sperm mixture was added to it. Then, 1 µL of the mixture was dropped on a Neobar slide and the sperms were counted.^   29 ^

**Evaluating** **sperm** **mobility. **A volume of 10 µL of sperm containing culture medium was placed on Neobar slide and covered with a cover-glass. Using a light microscope mobility of sperms was assessed at 10× and 20× magnification.^29^

**Evaluating viability of sperms.** For this purpose eosin-nigrosin staining was used. A volume of 20 µL of sperm sample was mixed with 20 µL eosin solution on a clean slide and after 20-30 sec, stained with 20 µL nigrosin solution. After preparing solution's smear and drying of slides , using an optical microscope the percentage of live (discolored) and dead (colored) sperms were analyzed.^   30^

**DNA strand damage level evaluation.** For assessing any kind of breakage in mice sperms DNA double strands, acridine orange staining was used. The percentage of spermatozoa stained with acridine orange was determined by counting 200 spermatozoa per slide. The monomeric acridine orange, bound to normal double-stranded DNA produces a green fluorescence, whereas the aggregated acridine orange on single-stranded DNA yields a yellow to red fluorescence. In this method after three times elution of sperm sample with PBS buffer, the precipitate was reached the final volume by PBS buffer. Desired smears of sperms culture medium was prepared and after being air-dried for 30 min was poured into a vessel containing the same ratios of acetone and ethanol. After that the slides were dried in air, placed in acridine orange solution for 7 min and after final drying were studied using a 100× fluorescence microscope and the results reported as percentage.^31^^,^^   32^

**Sperm nucleus maturity evaluation.** For this purpose aniline blue staining method was used. The basis of analysis is the replacement of protamine by histone in nucleus chromatin during spermiogenesis. This replacement has an important role in stability of sperm. In this staining method, immature sperms with high histone showed grayish dark blue in their nucleus and mature sperms were pale. Once again, after fixing sperm sample in acetone-ethanol solution and drying in air, slides were immersed in aniline blue solution for 7 min and after drying were examined by an optical microscope.^   33^

**Morphological evaluation of sperms.** In this study, aniline blue staining method was used and the percentage of abnormal sperms in appearance were recorded. Also, the eosin-nigrosin staining was used for more accurate observation. Specification of cytoplasmic residues of sperms were resulted from morphological immaturity. Those sperms with cytoplasmic residues were considered to be morphologically immature.^   34^

**Statistical analysis.** The data were analyzed by SPSS (Version 20; SPSS Inc., Chicago, USA) and one way ANOVA and Bonferroni test were used. A *p*-value less than 0.05 considered significant. 

## Results

**Fig. 1:**
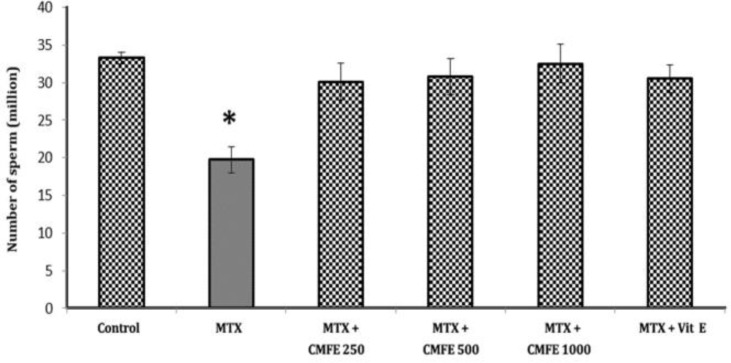
The average count of sperms in experimental groups. Asterisk indicates significant difference among the groups (*p* < 0.05).

**Average sperm count.** This study showed that there was a significant decrease in average number of sperms in the group receiving only MTX (19.7 ± 3.5) compared to other groups (*p* < 0.05), while other groups did not have any significant differences (Fig. 1).

**Fig. 2:**
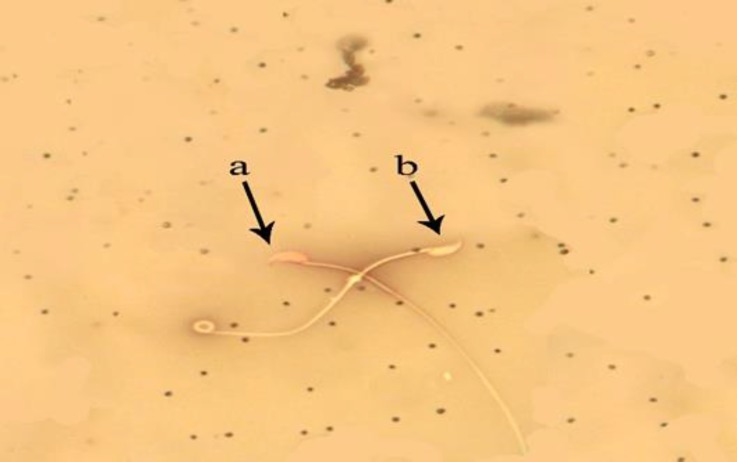
Evaluation of spermatozoa viability. **a)** Alive sperms have yellow heads (bright colored); **b)** Dead sperms have red heads (dark colored), (Eosin-nigrosin, 1000×).

**Viability power.** In MTX group a significant increase was observed in dead sperms compared to other groups except for group MTX + CMFE 250, (*p* < 0.05). However, the mean number of dead sperms in groups MTX + CMFE 500, MTX + CMFE 1000 and MTX + Vit E group were at the same level like the control group (Table 1 and Fig. 2).

**Sperm immobility status.** The average number of immobile sperms in groups MTX + CMFE 1000 (38.2 ± 4.1) and MTX + CMFE 500 (42.5 ± 5.0) remained similar to control group and no significant difference was observed. However, groups MTX, MTX + CMFE 250 (51.7 ± 2.6) and MTX + Vit E (52.2 ± 6.1) had significant differences with the control group (*p *< 0.05), (Table 1).

**Sperm morphology.** Any kind of disorder in sperm appearance, head, tail, and remaining cytoplasmic residues were considered as morphological disorders. The average number of sperms with unusual morphology was reported as percentages. There was a significant difference between groups MTX (41.0 ±5.5) and all groups except for MTX + CMFE 250 (42.0 ± 4.5) and MTX + Vit E (32.7 ± 4.0), (*p *< 0.05). 

**DNA damage.** DNA damage in sperms were calculated as percentage. Sperms with green nucleus were normal and orange to red ones (based on DNA damage) were considered with damaged DNA. Increasing of DNA damage in sperm, in MTX group (35.7 ± 6.9) was significantly higher compared to all other groups (*p* < 0.05), (Table 1 and Fig. 3). 

**Table 1:** The percentage of different parameters of sperm quality (Mean ± SE).

**Groups**	** Dead sperms**	**Immobile sperms**	** Abnormal sperms**	** DNA damaged sperms**	**Immature sperms**
**Control**	3.6 ± 4.3^a^	38.2 ± 3.7^a^	5.2 ± 2.2^a^	3.0 ± 8.1^a^	5.7 ± 2.8^a^
**MTX**	62.7 ± 5.4^c^	59.5 ± 8.9^c^	41.0 ± 5.5^c^	35.7 ± 6.9^c^	16.7 ± 3.6^c^
**MTX +** ** CMFE ** **1000**	40.0 ± 7.6^a^^b^	38.2 ± 4.1^a^	13.2 ± 2.6^a^	8.2 ± 1.5^a^	7.7 ± 3.5^a^^b^
**MTX +** ** CMFE ** **500**	45.0 ± 7.1^a^^b^	42.5 ± 5.0^a^^b^	27.2 ± 6.7^b^	8.5 ± 4.9^a^	7.7 ± 1.7^a^^b^^c^
**MTX +** ** CMFE ** **250**	50.7 ± 3.1^b^^c^	51.7 ± 2.6^b^^c^	42.0 ± 4.5^c^	18.5 ± 3.1^b^	15.0 ± 6.3^b^^c^
**MTX + Vit E **	44.5 ± 2.6^a^^b^	52.2 ± 6.1^b^^c^	32.7 ± 4.0^b^^c^	11.0 ± 0.8^a^^b^	7.7 ± 0.9^a^^b^^c^

abc Different letters in each column represent significant differences between groups (*p* < 0.05).

**Sperm nucleus immaturity. **Sperms with high histone in their nucleus showed grayish dark blue based on the histone level and considered as immature sperms. Significant increase was observed in average percentage of immature sperms in MTX group (16.7 ± 3.6) in comparison with all other groups except for MTX + CMFE 250 (15.0 ± 6.3) and MTX + Vit E groups (7.7 ± 0.9) (*p* < 0.05), (Fig. 4). 

**Fig. 5:**
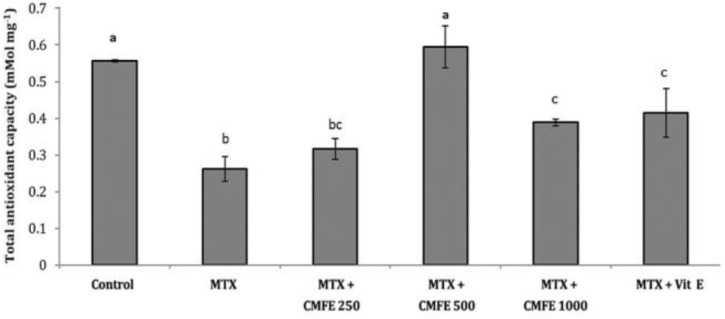
Profiles of total antioxidant capacity among the treatment groups. ^a,b,c^ Different letters indicate significant differences (*p *< 0.05).

In treatment groups which different doses of CMFE were used in combination with MTX, TAOC was significantly increased, especially in the treatment group which received 500 mg kg^-1^ CMFE (*p* < 0.05). The treatment group that received CMFE 500 mg kg^-1^ was not significantly different from the control group (Fig. 5).

**ab Fig. 3:**
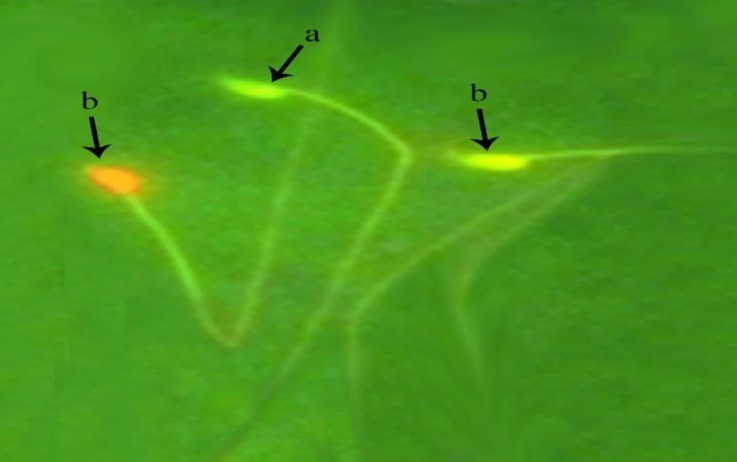
Detection of DNA damage in spermatozoa. **a)** DNA of healthy sperms have green heads; **b)** DNA of damaged sperms heads are in red color (Acridine orange, 1000×).

**b Fig. 4:**
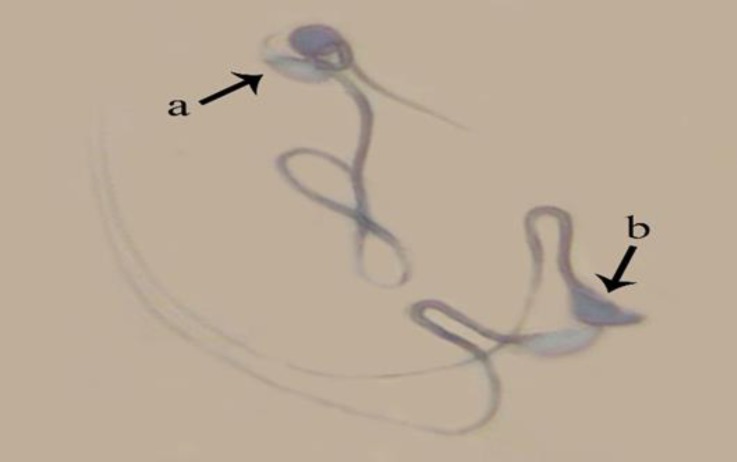
Maturity evaluation of spermatozoa. **a)** Sperms with mature nucleus show heads in pale blue; **b)** Immature nucleus sperms represent dark blue heads (Aniline blue, 1000×).

## Discussion

The results of this study revealed that MTX induced stress oxidative on cells of spermatogenesis was prevented with CMFE and Vit E. Mechanism of this protection is related to removing free radicals and annihilating them or increasing antioxidant capacity of body.^24^^,^^35^ Young and Love reported that most antioxidants act synergistically and create an effective barrier against oxidation.^   36 ^ Recent studies have shown that fruits and vegetables possess protective properties against diseases and CMFE is a rich source of antioxidants including flavonoids, anthocyanins and ascorbic acid.^   37 ^ These compounds protect proteins, lipids and DNA against free radicals.^   38 ^ Methotrexate is an anti-metabolic agent for a variety of neoplastic disorders and its toxic effects have been reported.^   39 ^ By acting on cell cycle, this agent prevents synthesis of DNA.^   9 ^ Similarity of MTX to folic acid helps it attach to DHFR. This enzyme is responsible for transformation of dihydrofolate to tetrahydrofolate. The latter compound acts as a mediator in synthesis of nucleotides (thymines and purines).^   40 ^ Regarding the mobility of sperms, the number of immobile sperms in groups MTX and MTX + CMFE 250 was increased in comparison with other groups. High energy requirements of sperms for movement explain why they possess many mitochondria. Increasing lipid peroxidation in mitochondria caused by methotrexate leads to degradation of mitochondrial membrane.^   35 ^ Therefore, antioxidants not only prevent decrease of the sperm mobility but also cause increase its capability.^   41 ^ Increasing the number of abnormal sperms is an indirect result of genotoxicity. Aziz *et al*. showed that there was a significant relationship between Reactive oxygen species (ROS) and increasing of malformed sperms (*p *< 0.05).

Disorders in morphology of sperms relates to spot mutations in reproductive cells^   34 ^ or chromosomal defects.^   42 ^ In this study the number of abnormal sperms in MTX group showed significant increase compared to other groups (*p* < 0.05) that completely complies with previous studies. Therefore, it can be said that sperm disorders induced by MTX due to physiological, cytotoxic and genetic changes in DNA cause an increase in the number of abnormal sperms.^   43 ^ In current study the average number of sperms in MTX group showed a significant decrease compared to other groups (*p* < 0.05). This reduction may be related to increased production of free radicals due to methotrexate administration that in turn causes disorders in functionality of Leydig and Sertoli cells.^44^^-^^46^ Since these cells play an important role in adjustment of testosterone levels, we can say that use of MTX causes a rise in ROS production slowing down spermatogenesis which would finally lead to a reduction in sperm count.^   47 ^ However, in other groups that received *cornus mas*, free radical production was limited and the number of sperms increased. In evaluation of viability, the mean number of dead sperms in group treated only with MTX compared to other groups was significantly high (*p *< 0.05), suggesting that this process may be related to positive peroxidase leukocytes in testis.^48^^,^^49^ However, decreasing the ratio of dead sperms in group that received CMFE may be associated with protective effect of CMFE which is an outcome of existing antioxidant compounds (like flavonoids etc.).^   50 ^ This study showed that the average number of sperms with damaged DNA in sham control group significantly increased in comparison to other groups, that it could be related to oxidative stress due to methotrexate.^   44 ^ There are three explanations about the causes of sperm DNA damage: 1) DNA damage is resulted from insufficient and inappropriate compaction and twisting of DNA in the period of maturing;^   51 ^ 2) Oxidative stress causes DNA damage;^   52 ^ and 3) Fragmentation of DNA caused by apoptosis.^   53 ^ In groups that received CMFE and Vit E, nucleotide DNA damage declined as a result of decreasing oxidative stress and free radicals. In the present study, it is revealed that percentage of immature (low quality chromatin) sperms in MTX+ CMFE 250 and MTX + Vit E groups were increased significantly in comparison with other groups (*p* < 0.05). In testis of mammals in the stage of spermiogenesis DNA-attached histones are replaced by basic proteins called protamine and this process consequently results in compaction of chromatin and stops the copying process. In epididymis phase protamin's thiol (-SH) groups are oxidized and inside their molecules disulfide bands are formed. Presence of these disulfide bridges is necessary for final compaction and physical/ chemical stability of chromatin. After desired compaction of chromatin, double strand DNA is surrounded by nucleo-protein and is protected against destroying factors like acids, heat, proteases, DNAase, ROS and detergents. It seems that disorders in chromatin compaction are associated with DNA and nucleus damage. In sperms whose nucleus are compacted weakly and replacement of protamine-histone is not completed, extensive DNA damages is expected. Most of these damage occur in the intermediate stage of spermiogenesis during protamine-histone replacement. As a result of deficiency in cytoplasmic antioxidants, sperms are not capable of repairing damages due to excess production of ROS.^52^^-^^54^ MTX increases the ROS production and it has been shown that the increased ROS production makes mitochondrial membranes highly susceptible to oxidative damage.^   55 ^ Based on the results of the present study, CMFE plays a role in decreasing oxidative stress by increasing the TAOC.

Therefore, it can be concluded that CMFE and Vit E could protect reproductive organs against MTX side effects. In this regards, CMFE with doses of 500 and 1000 mg kg^-1^ and Vit E (100 IU kg-1, po) had a good preventive effects against oxidative stress caused by MTX administration. 
